# Circulating LPS from gut microbiota leverages stenosis-induced deep vein thrombosis in mice

**DOI:** 10.1186/s12959-023-00514-2

**Published:** 2023-06-29

**Authors:** Cheng Liu, Ying Zhou, Huihui Gao, Zeping Zhang, Yu Zhou, Zifeng Xu, Chenhong Zhang, Zhen Xu, Huajun Zheng, Yan-Qing Ma

**Affiliations:** 1 Collaborative Research Program for Cell Adhesion Molecules, Shanghai University School of Life Sciences, Shanghai, China; 2 Department of General Surgery, School of Medicine, Ruijin Hospital, Shanghai Jiaotong University, Shanghai, China; 3 State Key Laboratory of Microbial Metabolism, School of Life Sciences and Biotechnology, Shanghai Jiaotong University, Shanghai, China; 4 Versiti Blood Research Institute, 8727 Watertown Plank Rd, Wisconsin, Milwaukee, WI 53226 USA; 5 NHC Key Lab of Reproduction Regulation, Shanghai Institute for Biomedical and Pharmaceutical Technologies, Fudan University, Shanghai, 200237 China; 6 Department of Biochemistry, Medical College of Milwaukee, Milwaukee, WI USA

**Keywords:** Deep venous thrombosis, Gut microbiota, LPS, TSP1, Prebiotics, Probiotics

## Abstract

**Objective and design:**

An accumulating body of evidence has shown that gut microbiota is involved in regulating inflammation; however, it remains undetermined if and how gut microbiota plays an important role in modulating deep venous thrombosis (DVT), which is an inflammation-involved thrombotic event.

**Subjects:**

Mice under different treatments were used in this study.

**Methods and treatment:**

We induced stenosis DVT in mice by partially ligating the inferior vena cava. Mice were treated with antibiotics, prebiotics, probiotics, or inflammatory reagents to modulate inflammatory states, and their effects on the levels of circulating LPS and DVT were examined.

**Results:**

Antibiotic-treated mice or germ-free mice exhibited compromised DVT. Treatment of mice with either prebiotics or probiotics effectively suppressed DVT, which was accompanied with the downregulation of circulating LPS. Restoration of circulating LPS in these mice with a low dose of LPS was able to restore DVT. LPS-induced DVT was blocked by a TLR4 antagonist. By performing proteomic analysis, we identified TSP1 as one of the downstream effectors of circulating LPS in DVT.

**Conclusion:**

These results suggest that gut microbiota may play a nonnegligible role in modulating DVT by leveraging the levels of LPS in circulation, thus shedding light on the development of gut microbiota-based strategies for preventing and treating DVT.

## Introduction

Deep veinous thrombosis (DVT) and pulmonary embolism are the two major manifestations of venous thromboembolism, collectively affecting 900,000 individuals in the US annually and leading to approximately 300,000 deaths, with half of survivals still suffering from post-thrombotic syndrome [1, 2]. DVT has been reported to be significantly high in critically ill patients with a high rate of recurrence [3, 4]. DVT has been ascribed to three major risk factors, venous stasis/stenosis, vascular wall injury, and hypercoagulability (Virchow’s Triad), but the involvement of other factors is still under exploration. Anticoagulation therapy is essential for treatment of DVT, but it carries a high risk of bleeding [5–7]. Therefore, prevention is the major focus for DVT, and there is an urgent need to further our understanding of the mechanistic regulation of DVT so that the development of more specific and safer strategies can be facilitated.

Gut microbiota plays an important role in influencing the overall health of the host [8, 9]. The ecosystem of gut microbiota is contingent on a variety of host factors, and dysbiosis of gut microbiota may lead to subacute or chronic inflammation, thus facilitating the development of multiple diseases, including cardiovascular disease, inflammatory bowel disease, and diabetes [10]. The influence of gut microbiota on inflammation can be modulated by leaking endotoxin lipopolysaccharides (LPS), which are components in the outer membrane of gram-negative bacteria, from the gut to circulation across intestinal epithelium [11]. LPS prime and activate innate immune cells via binding to TLR4 expressed on these cells, promoting the production of proinflammatory cytokines, such as TNF-α and IL-6 [12, 13]. LPS can also act on endothelial cells and platelets via TLR4 to facilitate the coagulation cascade [14, 15]. The leakage of LPS is primarily prevented by the tight junction structure of the intestinal epithelial barrier [11, 16], which can be protected by gut commensal microbes [17, 18]. Normally, gut commensal microbes greatly outnumber potential gram-negative pathogenic microbes to maintain a healthy gut environment [19]. Therefore, the intestinal barrier can be improved by either probiotics or prebiotics [20, 21]. In addition to endotoxins, some metabolites from gut microbes are also involved in modulating inflammation, such as trimethylamine N-oxide (TMAO). TMAO is generated in the liver of the host by converting trimethylamine (TMA), a metabolite of gut microbes when feeding with TMA-containing dietary nutrients [22], which potentially advances leukocyte recruitment and enhances platelet reactivity [23–25]. Hence, gut microbiota can participate in the regulation of inflammation via the translocation of endotoxin LPS and other metabolites into circulation.

Mechanistically, DVT has a feature that involves communication between the inflammatory response and the thrombotic reaction [26, 27], in which neutrophils and monocytes cooperate with platelets to initiate coagulation [28, 29]. Due to the important role of gut microbiota in inflammation, its influence on thrombosis has been observed in recent years. Thus far, multiple lines of research have established the connection between gut microbiota and arterial thrombosis [25, 30–32]. Although the involvement of gut microbiota in DVT has also been indicated in literature [33, 34], the precise role and the underlying mechanism remain unclear. In this study, we have examined the intrinsic effect of gut microbiota on the regulation of DVT in mice and disclosed a nonnegligible role of gut microbiota in stenosis DVT by modulating the levels of LPS in circulation.

## Materials and methods

### Experimental mice

Specific pathogen-free (SPF) C57BL/6 mice were purchased from Shanghai Legen Biotechnology (Shanghai, China) and housed under SPF conditions. Germ-free C57BL/6 mice were purchased from Cyagen Bioscience (Suzhou, China). Germ-free mice that underwent fecal microbiota transplantation (FMT) were housed in heterochronic donor cages containing soiled bedding and fecal pellets, and they received heterochronic donor microbiota via oral gavage. All animal studies have been approved by the Institutional Animal Care and Use Committee.

### Treatments of mice with antibiotics, prebiotics and probiotics

#### Antibiotic administration

C57BL/6 mice at six weeks of age were randomly divided into two groups. The treatment group was housed with access to drinking water containing an antibiotic cocktail (ABX: 1 mg/ml ampicillin sodium, 0.5 mg/ml vancomycin hydrochloride, 1 mg/ml neomycin sulfate, and 1 mg/ml metronidazole) for four weeks to eliminate intestinal bacteria, as previously described [35–37]. The control group of mice was housed with access to normal drinking water.

#### Prebiotic administration

C57BL/6 mice at six weeks of age were randomly divided into two groups. One group of mice was treated with xylooligosaccharides (XOS, 20 mg/kg body weight) by daily intragastric administration for four weeks. The control group of mice was treated with normal drinking water.

#### Probiotic administration

C57BL/6 mice at six weeks of age were randomly divided into two groups. One group of mice was treated with a probiotic cocktail including *Bifidobacterium*, *Lactobacillus* and *Enterococcus* (BLE, Shanghai Sine, 100 mg/kg body weight) by daily intragastric administration for four weeks. The control group of mice was treated with saline buffer.

### Treatment of mice with inflammatory reagents

#### Treatment of mice with LPS and LPS-RS

To test the effect of LPS on DVT, C57BL/6 mice at 8 weeks of age were treated with a low dose of LPS intravenously (Cat# L2880, Sigma-Aldrich; 50 ng/kg body weight) before the IVC ligation procedure. Some mice were pretreated with a TLR4 antagonist LPS-RS intravenously for three days (Cat# 13,106-MM, Invivogen; 1 mg/kg body weight/day). Mice in the control group were treated with sterile saline buffer.

#### Treatment of mice with TSP1 protein

Mice at eight weeks of age were randomized into two groups. Mice in the treatment group was intraperitoneally injected with purified TSP1 protein (Cat# CU45, Novoprotein; 2.5 mg/kg body weight) before the IVC ligation procedure. Mice in the control group were injected with sterile saline buffer.

### Stenosis-induced DVT model

Mice were deeply anesthetized by continuous isoflurane-oxygen inhalation. In brief, mice were placed in a transparent induction chamber with a precision calibrated vaporizer set to the inducing dose of isoflurane (5%). The unconscious mice were then transferred to a warm surgical surface equipped with a nose cone connected to the vaporizer to maintain deep anesthesia at a dose of isoflurane (2–3%), assessed by toe pinch withdrawal and respiratory rate. A laparotomy was performed to expose the inferior vena cava (IVC) after carefully separating it from the surrounding tissues near the renal veins. The IVC was ligated over a spacer using a 5.0 monofilament polypropylene filament. Following ligation, the spacer was carefully removed to avoid complete vessel occlusion. Additionally, branches were either ligated or cauterized. The peritoneum and skin were immediately closed and sutured. At defined time points, mice were euthanized, and the IVC tissues were collected for measurement. Based on our preliminary studies, the prevalence of DVT in the IVC of control C57BL/6 mice ranged from 10 to 30% after partial ligation for 6 h. The prevalence increased to approximately 70 ~ 80% after partial ligation for 24 h. Therefore, these two time points were chosen to evaluate factors promoting or inhibiting DVT. All mice were assigned unique numbers, and the IVC ligation procedure was performed in a blinded fashion.

### Collection and analysis of blood samples

#### Plasma preparation

Blood samples was collected from the facial vein using tubes containing either EDTA or heparin as anticoagulants. The collected whole blood was centrifuged at 1,200 g for 10 min to obtain plasma samples.

#### Endotoxin measurement

The concentration of endotoxin in plasma was measured using the Limulus Amebocyte Lysate (LAL) assay with using a commercial kit (Cat# EC32545S, Xiamen Hou Reagents, China).

#### Quantification of TMAO

Serum samples were prepared precipitating proteins using a solvent mixture of acetonitrile and water (7:3) followed by sonication for 15 min. After centrifugation, the supernatants were transferred to new polypropylene tubes for analysis. The quantification of TMAO was performed using LC-MS/MS with a UPLC system (Waters) coupled to a QTRAP6500 mass spectrometer (SCIEX, USA). A Waters BEH HILIC chromatographic column (100 mm × 2.1 mm, 1.7 μm) was used, and the ion source was ESI, operating in positive ion mode. The obtained results were analyzed using MultiQuant™ 3.0.2 Software.

#### Plasma proteome analysis

Quantitative proteomic analysis was performed using the iTRAQ (isobaric Tags for Relative and Absolute Quantitation) technology. High abundant proteins in plasma samples were first immunodepleted. Solution IEF (Isoelectric Focusing) was employed as a second fractionation step to reduce the complexity of plasma proteome. Following quantification, 100 µg of protein sample was subjected to trypsin proteolysis, and the resulting peptides were labeled using the iTRAQ Reagent 8-plex kit (Applied Biosystems). Fractionation of the labeled peptide mixture was achieved by SCX (strong cation exchange) chromatography with an LC-20AB HPLC pump system (Shimadzu, Kyoto, Japan). Each fraction was reconstituted in phase A (2% acetonitrile and 0.1% formic acid), centrifuged, and the peptides in supernatant were isolated using an UltiMate 3000 UHPLC (Thermo Fisher Scientific, San Jose, CA). The isolated peptides were ionized by nanoESI source and analyzed by a benchtop orbitrap Q-Exactive™ HF-X mass spectrometer (Thermo Fisher Scientific, San Jose, CA) coupled online to the UHPLC. Raw data files acquired from the Q Exactive instrument were converted into MGF (Mascot Generic File) format using Proteome Discoverer version 1.4 (Thermo) and the MGF files were searched against mouse protein sequences from the UniProt database using Mascot 2.3.02 (Matrix Science). The precursor mass tolerance was set at 10 ppm, and the product ion tolerance was set at 0.05 Da. False discovery rates (FDRs) were obtained using Percolator, and only identification with a q-value equal to or less than 0.01 were considered. Protein quantification was based on unique peptides. The iTRAQ quantitation was processed using the software IQuant [38]. The upregulated and the downregulated proteins in the LPS-treated and XOS-treated samples, compared to their respective controls, were identified. Proteins with ratios ≥ 1.20 or ≤ 0.83, and a *P*-value < 0.05, were considered as statistically significant.

### 16 S rRNA gene sequencing and data analysis

For the analysis of gut microbiota, 16s rRNA gene sequencing was performed. The genomic DNA was extracted from the fecal samples using the QIAamp PowerFecal Pro DNA Kit (Qiagen, USA). The V3-V4 region of the 16S rRNA gene was amplified using the primers 338-F (5’- CCTACGGGNGGCWGCAG-3’) and 806-R (5’- GACTACHVGGGTATCTAATCC-3’) with TransStart Fastpfu DNA Polymerase (TransGen, Beijing, China) for 20 cycles. The amplicons were purified using the QIAquick PCR Purification Kit (Qiagen) and pooled at equal concentrations. The pooled amplicons were then subjected to sequencing on an Illumina MiSeq instrument (Illumina, San Diego, CA, USA) using a 2 × 300 cycle run. Quality filtering and clustering to the operational taxonomic units (OTUs) at a 97% similarity level of the 16 S rRNA gene sequences were carried out using Mothur software (version 1.41.1). The classification of rRNA gene sequences was performed using the SILVA SSU Ref database (v132, 99%). Based on the classification information of samples (phylum), a heatmap of fecal microbiota from mice and the clustering relationship of the samples were constructed. The linear discriminant analysis (LDA) effect size (LEfSe) method was utilized to identify bacterial taxa that were significantly different enriched in each group, with an LDA score > 2.5. Alpha diversity was statistically analyzed using one-way analysis variance (ANOVA).

### Histological study

Freshly collected intestinal tissues were fixed with 4% PFA and subjected to standard histological process, including embedding them in paraffin and obtaining 3 μm sections. For IHC staining, the tissue sections were stained with an anti-occludin antibody (Cat# GB111401, Servicebio, Wuhan) or an anti-ZO-1 antibody (Cat# GB11195, Servicebio, Wuhan) after antigen retrieval. The IHC images were taken under a microscope (Nikon E100), and the IHC staining intensity were further quantified based on optical density.

### Western blotting

Extracted proteins were quantified using a BCA Protein Assay kit (Cat# 23,227, Thermo Fisher Scientific), and the samples were loaded onto SDS-PAGE using 4–20% gradient gels (Cat# M00656, GenScript Biotech), followed by transfer to a PVDF membrane (Cat# ISEQ00010, Merck Millipore). After the transfer, the membrane was blocked with QuickBlock™ Western Blocking Buffer (Cat# P0252, Beyotime Biotech) for 1 h at room temperature and then used for immunoblotting. Antibodies used in study included an anti-occludin antibody (Cat# ab167161, Abcam), an anti-ZO-1 antibody (Cat# 21773-1-AP, Proteintech), and an anti-β-actin antibody (Cat# 66009-1-1 g, Proteintech), an anti-TSP1 antibody (Cat# sc-59,887, Santa Cruz Biotechnology), and an anti-transferrin antibody (Cat# ab278498, Abcam).

### Statistical analysis

Results are expressed as means ± SD. *P* values were calculated using the two-tailed Student’s *t* test. One-way ANOVA with post hoc tests was performed for multiple comparisons. A probability level of *p < 0.05* was considered statistically significant.

## Results

### ABX treatment or germ depletion effectively suppresses DVT in mice

**Fig. 1:**
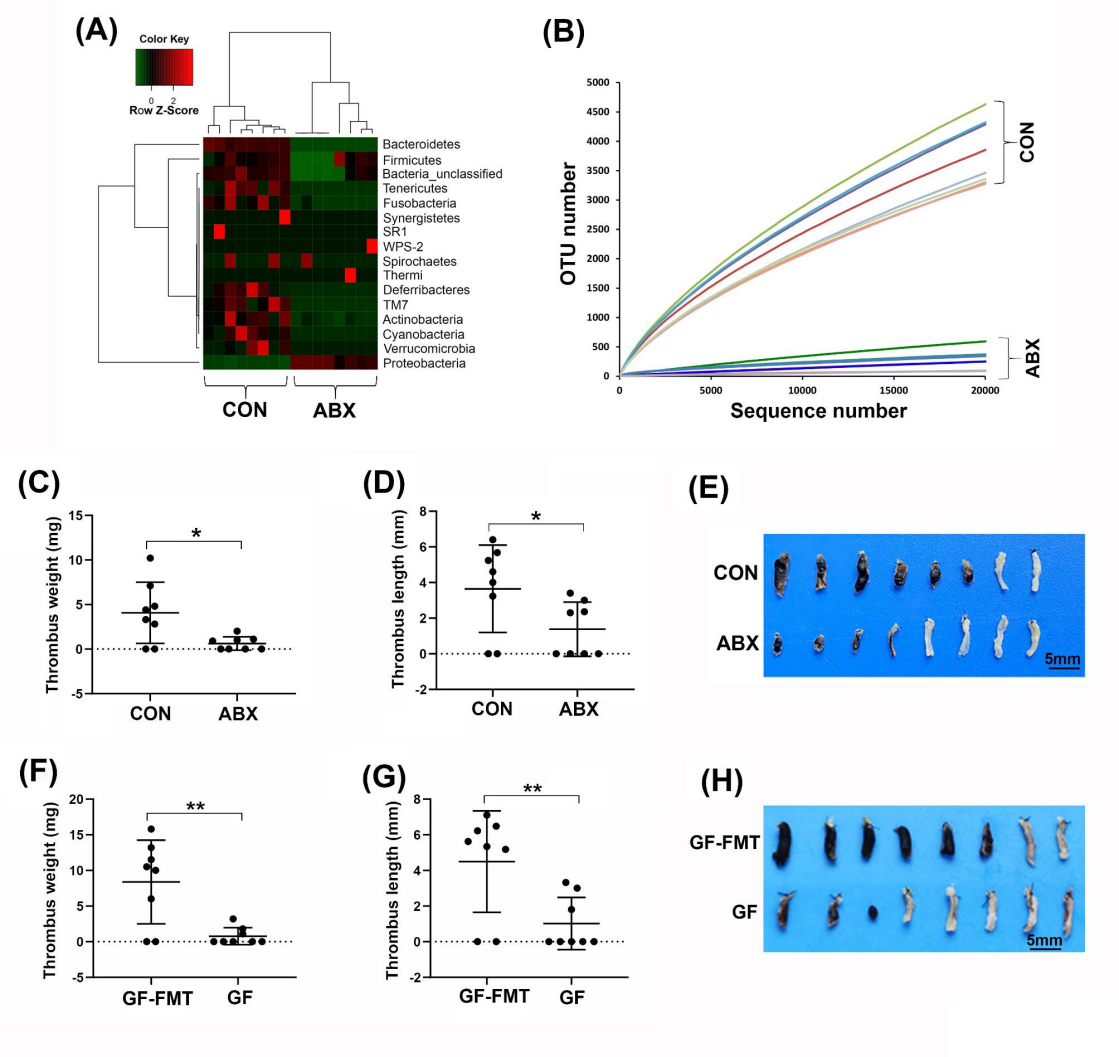
Germ depletion in mice suppresses DVT. (**A**) The heatmap shows the fecal microbiome of control (CON) and antibiotic (ABX)-treated mice based on 16 S rRNA gene sequencing data (n = 8). (**B**) Rarefaction curves were calculated at the 97% similarity level with 16 S rRNA gene sequencing data from the microbiota of CON and ABX-treated mice. (**C**-**E**) Partial ligation of the inferior vena cava (IVC) was performed on CON (n = 8) and ABX-treated mice (n = 8) to induce DVT. After 24 h of ligation, the IVC tissues were harvested, and the formed thrombi were quantified and imaged. (**F**-**H**) Partial ligation of the inferior vena cava (IVC) was performed to induced DVT in germ-free mice (GF, n = 8) or GF mice with restored gut microbiota by FMT (GF-FMT, n = 8). After 24 h of ligation, the IVC tissues were harvested, and the formed thrombi were quantified and imaged. Data are shown as mean ± SD. (*, *p < 0.05*; **, *p < 0.01*)

To investigate the contribution of gut microbiota to the development of DVT in mice, we treated mice with a broad-spectrum ABX cocktail to deplete the gut microbiota, as previously described [36]. Following the treatment, a general reduction in the relative abundance of a cluster of phyla in the feces of the ABX-treated mice was observed (Fig. 1, A and B). DVT was induced in both the ABX-treated mice and control mice by partially ligating the IVC, as we previously described [29]. Interestingly, we found that DVT was significantly suppressed in the ABX-treated mice compared to the untreated control mice (Fig. 1, C-E), indicating the potential involvement of gut microbes in facilitating stenosis-induced DVT. Furthermore, we utilized germ-free mice and observed that DVT was also suppressed in these mice compared to germ-free mice after fecal microbiota transplantation (FMT) (Fig. 1, F-H), providing further evidence that gut microbiota may play a role in promoting DVT.

### Prebiotic or probiotic treatment suppresses DVT in mice

**Fig. 2:**
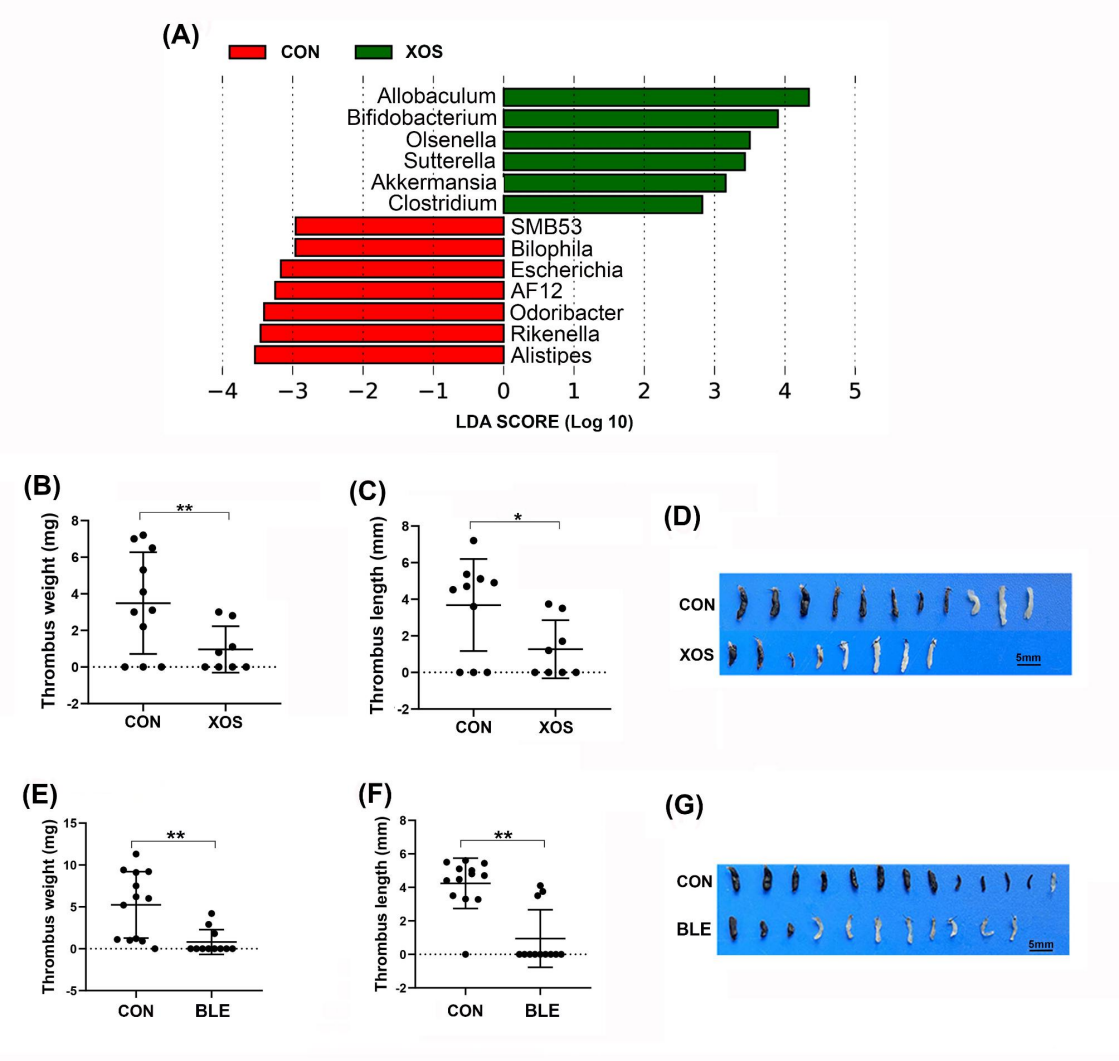
Treatment of mice with prebiotics or probiotics suppresses DVT. (**A**) The alteration of fecal microbiome was analyzed between control (CON) and XOS-treated mice. The bar graph shows the LDA scores, with green bars indicating genus enriched in the XOS group and red bars indicating genus enriched in the CON group. (**B**-**D**) The partial IVC ligation procedure was performed after feeding mice with xylooligosaccharides (XOS, n = 8) for 4 weeks. After 24 h, the IVC tissues were harvested, and the formed thrombi were quantified and imaged, and the results were compared to those formed in CON mice (n = 11). (**E**-**G**) The partial IVC ligation procedure was performed on mice treated with a probiotic cocktail including *Bifidobacterium*, *Lactobacillus* and *Enterococcus* (BLE, n = 11), or saline buffer as CON (n = 13) for 4 weeks. After 24 h, the formed thrombi in the IVC tissues of these mice were quantified and imaged. Data are presented as mean ± SD. (*, *p < 0.05*; **, *p < 0.01*)

Commensal microbes play an essential role in maintaining a diverse ecosystem of gut microbiota by suppressing pathogenic microbes [19]. The health of the gut microbiota can be nurtured by use of prebiotics or probiotics [20, 21]. XOS are known prebiotics to promote the growth of beneficial commensal bacteria, such as *Bifidobacterium*, in the gut and enhance the gut barrier function [39, 40]. As expected, we observed a significant increase in the abundance of *Bifidobacterium* in mice fed with XOS (Fig. 2A). Importantly, we found that feeding mice with XOS significantly suppressed DVT (Fig. 2, B-D). Additionally, when we directly fed mice with probiotics containing *Bifidobacterium*, *Lactobacillus* and *Enterococcus*, we also observed a suppression of DVT (Fig. 2, E-G). These results indicate that enrichment of commensal bacteria through use of either prebiotics or probiotics can efficiently reduce the risk of DVT. Taken together with the findings from ABX-treated or germ-free mice, these observations suggest that an imbalance in pathogenic microbes in the gut may contribute to the promotion of DVT in mice.

### Circulating LPS derived from gut microbiota plays an important role in leveraging DVT in mice

**Fig. 3:**
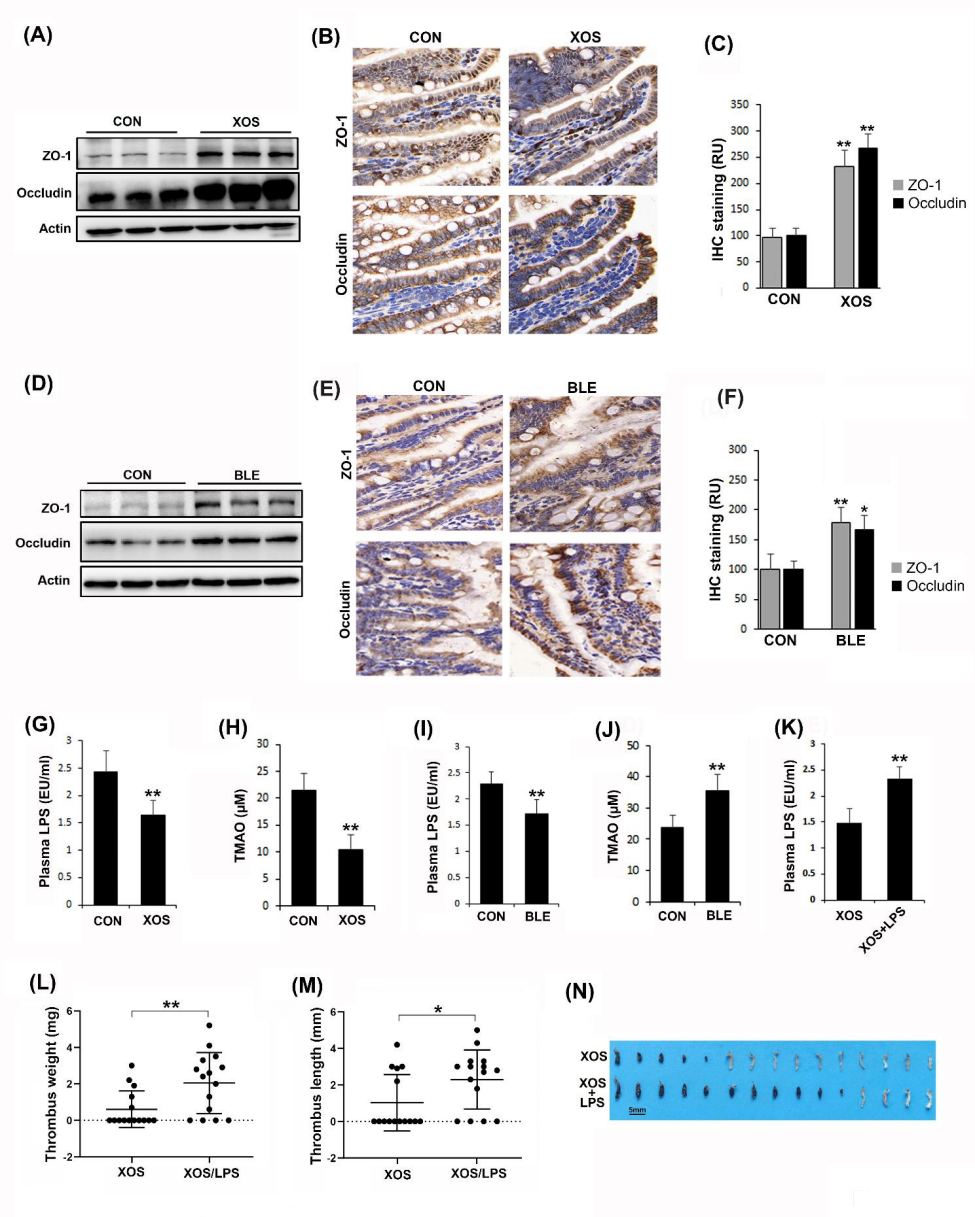
Treatment of mice with prebiotics or probiotics enhances the gut barrier and reduces the levels of circulating LPS. (**A**-**C**) Intestinal tissues were collected from control (CON) and XOS-treated mice. The expression levels of tight junction markers (Occludin and ZO-1) were examined using both Western blotting (**A**) and histological IHC staining (**B** & **C**). (**D**-**F**) Intestinal tissues were collected from CON and BLE-treated mice. The expression levels of tight junction markers (Occludin and ZO-1) were examined using both Western blotting (**D**) and histological IHC staining (**E** & **F**). IHC images were captured using a 40× objective. (**G** & **H**) The levels of LPS and TMAO in plasma of CON and XOS-fed mice were quantified by the limulus amebocyte lysate assay and LC-MS/MS, respectively. (**I** & **J**) The levels of LPS and TMAO in plasma of CON and BLE-treated mice were quantified by the limulus amebocyte lysate assay and LC-MS/MS, respectively. (**K**) Mice were fed with XOS and then the levels of LPS were restored by treating mice with a low dose of LPS (50 ng/kg). (**L**-**N**) The partial IVC ligation procedure was performed on mice fed with XOS with or without LPS treatment. After 6 h, the formed thrombi in the IVC of these mice were quantified, imaged, and compared (n = 15). Data are presented as mean ± SD. (*, *p < 0.05*; **, *p < 0.01*)

To explore the possible mechanism involved, we examined whether treatment of mice with prebiotics or probiotics affects the levels of circulating LPS and TMAO in circulation since these are known inflammatory factors originated from the gut microbiota. Since the gut barrier provides a physical and functional barrier against the transport of LPS from the gut to circulation, we first examined the expression levels of intestinal tight junction proteins, including occludin and ZO-1, in the intestinal epithelium. We observed that treating with either XOS (Fig. 3, A-C) or BLE (Fig. 3, D-F) significantly enhanced the expression levels of these tight junction proteins. Consistently, both treatments resulted in reduced levels of LPS in circulation (Fig. 3, G and I). Interestingly, treatment of mice with XOS led to decreased levels TMAO, while treatment with BLE unexpectedly increased the levels of TMAO (Fig. 3, H and J). These findings suggest that LPS in circulation derived from the gut microbiota may play a more potent role in affecting DVT compared to TMAO. To verify the importance of LPS in DVT, we restored the levels of circulating LPS in XOS-treated mice to similar levels as as untreated control mice by injecting a low dose of LPS (50 ng/kg) (Fig. 3, G and K). We found that stenosis-induced DVT was restored in these mice (XOS + LPS) (Figs. 2 and B-D and 3 and L-N). Similarly, elevating the levels of circulating LPS in BLE-treated mice also restored DVT (data not shown). Collectively, these results demonstrate that the levels of circulating LPS derived from the gut may play an important role in exacerbating DVT in mice.

### LPS promotes DVT via TLR4

**Fig. 4:**
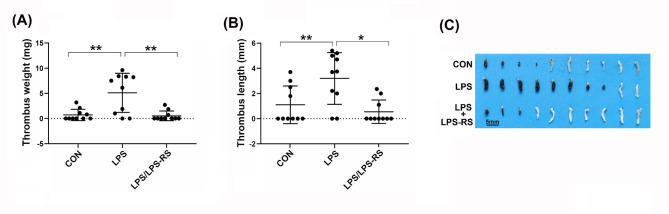
Elevation of circulating LPS in mice promotes DVT via binding to TLR4. (**A**-**C**) Mice were treated with a low dose of LPS (50 ng/ml) with or without pretreatment of a TLR4 antagonist. Untreated mice served as controls (CON). The partial IVC ligation assay was performed on these mice. After 6 h, the IVC tissues were collected, and the formed thrombi in the IVC were quantified and imaged (n = 10). Data are shown as mean ± SD. (*, *p < 0.05*; **, *p < 0.01*)

As is known, LPS can initiate potential inflammatory responses by binding to TLR4 on innate immune cells [41, 42]. To further test the involvement of LPS-TLR4 signaling in DVT, we treated mice with a low dose of LPS (50 ng/kg) in the presence or absence of LPS-RS, a potent TLR4 antagonist [43]. We observed that treatment of mice with LPS significantly enhanced DVT, while pretreatment of mice with the TLR4 antagonist LPS-RS completely blocked the enhancement of LPS-induced DVT (Fig. 4, A-C). These findings suggest that LPS promotes DVT through the LPS-TLR4 signaling pathway.

### Identification the downstream effectors of LPS in DVT

**Fig. 5:**
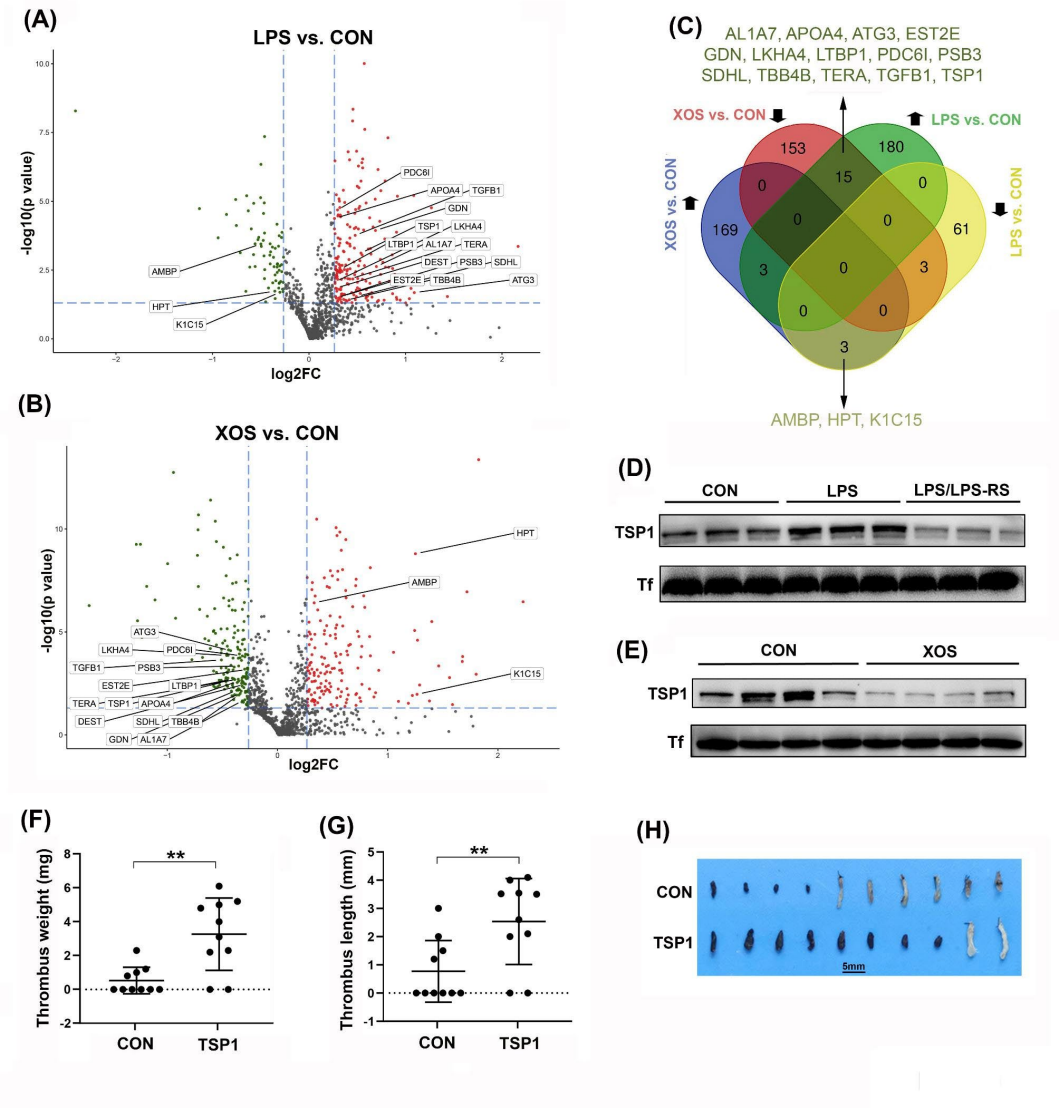
Identification of protein downstream effectors of LPS in plasma for DVT in mice. Quantitative proteomics was performed to quantify plasma proteins in LPS-treated and XOS-treated mice, and their controls (CON). (**A** & **B**) Volcano plots illustrate the upregulated (red dots) and downregulated (green dots) proteins in LPS-treated and XOS-treated samples when compared to their controls. (**C**) The plasma proteins that were reversely regulated in LPS-treated and XOS-treated groups are listed. (**D** & **E**) Western blotting was conducted to compare TSP1 levels in the plasma of treated mice and their controls. (**F**-**H**) Mice were intravenously injected with either TSP1 or saline buffer (CON), followed by the partial IVC ligation for 6 h. The IVC tissues were collected and the formed thrombi in the IVC were quantified and imaged (n = 10). Data are shown as mean ± SD. (**, *p < 0.01*)

To identify possible downstream effectors of circulating LPS in DVT, iTRAQ quantification proteomics was employed to examine altered plasma proteins in LPS-treated mice. We discovered that 198 proteins were upregulated, while 67 proteins were downregulated in the plasma of LPS-treated mice compared to the controls. Additionally, we examined altered proteins in the plasma of XOS-treated mice, as they exhibited the opposite effect on DVT. In XOS-treated mice, we observed that 175 proteins were upregulated, and 171 proteins were downregulated compared to the controls. To narrow down the effector candidates, we focused on plasma proteins with opposite alterations between LPS-treated mice and XOS-treated mice. As a result, we identified 15 proteins that were upregulated after LPS treatment and downregulated after XOS treatment, and 3 proteins that were reversely altered in these mice (Fig. 5, A-C).

Of particular interest, TSP1 was one of the plasma proteins found to be upregulated in LPS-treated mice and downregulated in XOS-treated mice (Fig. 5C). TSP1 is a major component of platelet α-granules and is secreted upon platelet activation, potentially playing a role in promoting hemostasis [44–46]. It is also highly expressed in myeloid lineage cells and functions in innate immunity in conjunction with its ligand CD47 [47, 48]. In this study, we focused on TSP1 and examined its potential role in DVT, which has not been previously investigated. Western blotting analysis confirmed its upregulation in the plasma of LPS-treated mice and its downregulation in XOS-treated mice (Fig. 5, D and E). Importantly, the upregulation of TSP1 induced by LPS was blocked by the TLR4 antagonist LPS-RS (Fig. 5D), suggesting that LPS-induced upregulation of TSP1 in circulation is mediated by the LPS-TLR4 signaling pathway. Furthermore, treatment of mice with purified TSP1 protein promoted DVT (Fig. 5, F-H). These results indicate that TSP1 may serve as an effective downstream effector of LPS in promoting stenosis-induced DVT in mice. However, we also observed that Serpine2 (GDN), a known anticoagulant protein [49], exhibited similar regulation to TSP in these mice (Fig. 5, A-C). This suggests that the role of circulating LPS in modulating DVT could be balanced by multiple downstream effectors in plasma, and their integrated effect may ultimately determine the status of DVT. Therefore, it is intriguing to investigate the involvement of these altered proteins and their collaborative role in regulating DVT in future studies.

## Discussion

In this study, we have demonstrated the involvement of gut microbiota in the regulation of stenosis-induced DVT. Firstly, we observed that DVT was suppressed in mice treated with a broad-spectrum ABX or in germ-free mice. Secondly, treatment of mice with prebiotics or probiotics, which are effective in enhancing commensal bacteria in the gut, resulted in a compromised ability to develop DVT. These findings suggest that pathogenic bacteria in the gut microbiota may act as promoters of DVT. It is known that gut microbiota can modulate systemic inflammation by altering the levels of circulating LPS and TMAO. Importantly, application of prebiotics or probiotics efficiently reduced the levels of circulating LPS in mice. Additionally, restoring the circulating LPS levels in these mice by injecting LPS reinstated the ability to develop DVT. These findings imply that the levels of circulating LPS derived from the gut microbiota may play a role in modulating stenosis-induced DVT. It is worth noting that a very low dose of LPS (50 ng/kg) was used to retore the circulating LPS levels to those observed in untreated mice, which is significantly less than previously reported sublethal dose (2 mg/kg) [50]. We believe that these findings are important because they suggest that downregulating circulating LPS by maintaining a healthy gut microbiota ecosystem may help reduce the risk of DVT. In addition to LPS, other pathogen-associated molecular patterns from the gut microbiota may also participate in the regulation of DVT. For instance, it has been reported that peptidoglycan translocated from the gut to circulation has the potential to promote platelet aggregation, prime systemic innate immunity, and induce disseminated intravascular coagulation [51–53].

Surprisingly, we observed a reduction of circulating TMAO only in mice treated with prebiotics (XOS), but not those treated with probiotics (BLE), despite both treatments leading to a reduction of circulating LPS and the suppression of DVT. These observations suggest the anti-DVT effect of prebiotics or probiotics may be associated with multiple factors derived from the gut microbiota, among which LPS may be a potent one.

Innate immune cells, platelets, and local vascular endothelial cells are all involved in initiation and progression of DVT [28, 54]. Since a TLR4 antagonist effectively blocks LPS-induced DVT, it is likely that the promoting effect of LPS on DVT occurs through binding to TLR4 on these cells and triggering downstream inflammatory responses [12–15, 55]. To further explore possible downstream effectors of circulating LPS in DVT, we conducted proteomic analysis to examine altered plasma proteins in mice after LPS treatment. Additionally, we included XOS-treated samples in the analysis since XOS treatment showed an opposite effect on DVT. Our results revealed that TSP1 is among the plasma proteins upregulated by LPS treatment and downregulated by XOS treatment. Importantly, we demonstrated that TSP1 has the ability to promote DVT, suggesting that it may serve as one of the downstream effectors of LPS in inducing DVT. Previous studies have reported that TSP1 released by platelets is required for hemostasis by binding to CD36 on platelets [45]. TSP1 also plays a role in innate immunity by interacting with its ligand CD47 on myeloid lineage cells and endothelial cells [47, 48, 56, 57]. Therefore, it would be interesting to further explore how TSP1, along with the other altered proteins, contribute to the modulation of DVT in future studies.

## Data Availability

The data supporting the findings of this study are available within the article and its supplementary materials.
